# Taxonomic composition and predicted functional potential of the sputum bacterial microbiome in Iraqi patients with bronchiectasis using 16S rRNA gene amplicon sequencing

**DOI:** 10.1016/j.jgeb.2026.100810

**Published:** 2026-09-07

**Authors:** Maryam Hadi, H. Halah Al-Haideri

**Affiliations:** a University of Mustansiriyah College of Dentistry, Baghdad, Iraq; b University of Baghdad College of Science for Women, Baghdad, Iraq

**Keywords:** Airway microbiome, Amplicon sequence variant, Bronchiectasis, 16S rRNA gene, Sputum microbiome, Microbial diversity, Taxonomic composition

## Abstract

Bronchiectasis is a chronic respiratory disease characterized by persistent airway inflammation and recurrent infections. Although alterations in the airway microbiome have been reported, data from Middle Eastern populations remain limited. This study aimed to characterize the sputum bacterial microbiome and predict its functional potential in Iraqi patients with bronchiectasis using 16S rRNA gene (V3-V4) amplicon sequencing. Sputum samples were collected from 28 patients with non-cystic fibrosis bronchiectasis and 10 healthy individuals. Amplicon sequence variants (ASVs) were inferred using DADA2, taxonomic assignment was performed against the SILVA (v138.2) database, and alpha and beta diversity were assessed. Differential abundance was analysed using ALDEx2, and predicted functional profiles were inferred using Tax4Fun2 based on KEGG pathways. Taxonomic profiling identified 24 bacterial genera across seven phyla. Sputum microbiota was dominated by *Pseudomonas*, *Streptococcus*, *Neisseria*, and *Haemophilus*, with descriptive differences in the relative abundance of several genera between bronchiectasis and healthy controls. However*,* no significant differences were observed in alpha diversity, beta diversity, differential abundance after Benjamini-Hochberg correction, or predicted KEGG pathway abundances between groups in Iraqi patients with bronchiectasis using 16S rRNA gene amplicon sequencing. Although compositional trends were observed, no statistically significant differences in microbial diversity, differential abundance, or predicted functional pathways were detected. These findings provide a baseline for future larger, longitudinal studies investigating the airway microbiome in bronchiectasis.

## Highlights

• The sputum bacterial microbiome of Iraqi bronchiectasis patients was characterized using 16S rRNA gene (V3-V4) amplicon sequencing.
• Twenty-four bacterial genera representing seven phyla were identified, with *Pseudomonas*, *Streptococcus, Neisseria*, and *Haemophilus,* as the dominant genera.
• The alpha diversity, beta diversity, differential genus abundance after multiple-testing correction, or predicted KEGG pathways indicated no significant differences between groups.

## Graphical abstract

**Figure:**
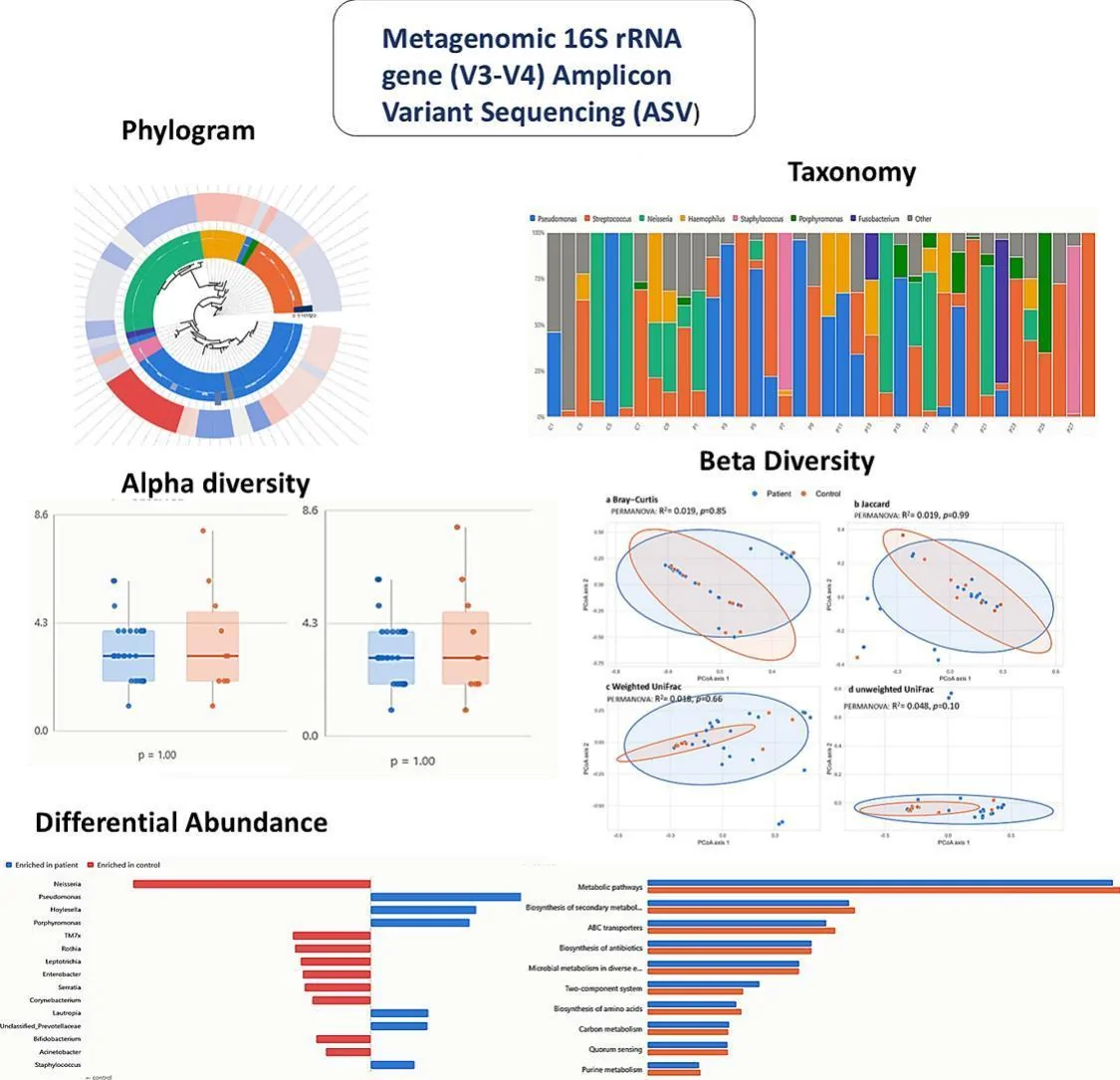

## 1 Introduction

Bronchiectasis is a chronic respiratory disease with heterogeneous clinical manifestations and outcomes.^1^, ^2^ Worldwide, the prevalence has risen sharply; it was reported to be around 566 per 100.000 population in Europe and North America, and as high as 1200 per 100.000 population in China, reflecting the growing and costly public health burden.^3^ Bronchiectasis is characterized by a complex interaction between chronic infection, airway inflammation, and attenuation of mucociliary clearance, resulting in irreversible airway dilatation, increased airway wall thickness, and chronic productive cough. The interaction of one factor with another exacerbates the disease.^4^ Chronic bacterial infection sustains neutrophilic inflammation by the release of neutrophil elastases and neutrophil extracellular trap formation mechanisms, which preserve the vicious vortex of infection and inflammation and structural airway damage. This pathophysiology was first described by Flume, Chalmers and Oliver, and then defined as an interlinked infection-microbiome-inflammation cycle.^5^, ^6^

The lack of approved bronchiectasis-specific pharmacotherapy targets the cystic fibrosis-associated disease; management based on long-term antibiotic treatment is one of the most common therapies targeting chronic pathogens, such as *Pseudomonas aeruginosa,* which is one of the most common pathogens associated with bronchiectasis in culture.^7^ These therapeutic challenges have increased interest in understanding the airway microbiome and its contribution to disease progression.

The airway microbiome comprises diverse microbial communities; the composition of these communities is influenced by host and environmental factors and has an important role in respiratory health and disease.^8^, ^9^ The microbial composition of the lung was investigated *in vitro* (in culture); however, this method detects the growth of known aerobic organisms; therefore, alternative methods, such as next-generation sequencing, particularly 16S rRNA gene amplicon sequencing, have improved the characterization of bronchiectasis airways beyond what can be achieved using conventional culture methods.^3^, ^5^ Therefore, they provide new insights into the exact interrelationships between microbiota alteration and disease progression, and into detecting host–microorganism interactions.^9^, ^10^, ^11^

Several studies have characterized the airway microbiome of patients with bronchiectasis, including bacteria, fungi, mycobacteria, and viral communities; however, conventional microbiological methods incompletely capture the diversity of airway microorganisms. Several factors can influence the microbiome composition in the airway, such as the genetic environment, lifestyle, and geographical region, which may vary according to different ethnic groups.^12^ The alteration in the composition of sputum microbiota in bronchiectasis was revealed using 16S rRNA gene amplicon sequencing by the studies of MacAogain et al. and Richardson et al., which showed that the microbiome composition differs markedly from that in the sputum microbiota of healthy people.^3^, ^5^ They reported that Pseudomonadota (Proteobacteria) was the most dominant phylum in bronchiectasis, followed by Bacillota (Firmicutes), and dominant genera such as *Pseudomonas, Haemophilus, Streptococcus, and Veillonella,* whereas the healthy sputum was enriched mainly by *Prevotella* and *Veillonella.*^3^, ^5^

In some severe cases, the airway microbiome may become dysbiotic, frequently due to the prevalence of a single pathogenic taxon such as *P. aeruginosa.* In a cohort study, the sputum microbiome profile of 281 patients was dominated by *Pseudomonas spp* and associated with a threefold increase in mortality and exacerbation in comparison to other microbiome profiles.^13^ Likely, the BLESS trial cohort analysis revealed that the microbiota dominated by *Haemophilus* or *Pseudomonas* resulted in a reduction in lung function and exacerbated systemic and airway inflammatory markers; the *Haemophilus*-dominant profile is frequently linked with fewer exacerbations than a *Pseudomonas* or *Veillonella* dominance.^3^, ^14^

Reduced microbial diversity, reflected by lower richness or evenness, has been associated with greater disease severity, accelerated lung function decline, and increased mortality.^6^, ^14^, ^15^ A longitudinal study revealed that the bacteriome profiles of individuals exposed to antibiotics remain significantly unchanged over many years and establish dysbiosis rather than a transient state.^3^, ^15^ Many studies have been established with mycobiome and virome, revealing that some fungi such as *Aspergillus fumigatus* and *A. terreus*, and some respiratory viruses also interact with the bacterial community, and contribute to exacerbation and immune dysregulation; however, little information is available about their association.^3^, ^16^

Despite this progress, important gaps in the bronchiectasis microbiome literature remain. Several published studies were based mainly on 16S rRNA amplicon sequencing to describe taxonomic composition, without assessing the functional or metabolic capacity of the airway microbiota.^5^, ^6^ The predicted functional potential has been reported in a small number of cohort studies; therefore, the functional consequences of bronchiectasis-associated dysbiosis remain less well characterized than its taxonomic composition.^16^ Furthermore, many studies have been conducted for European, North American, or East Asian populations, with very few studies from the Middle East. To our knowledge, no studies have been reported for the Iraqi population; however, the geographical variation in airway microbiome composition, aetiology, and microbiology of bronchiectasis has been well characterized.^3^, ^16^ Lack of regional data limits the interaction between the findings from other populations and leaves the specific microbial and functional signatures of bronchiectasis in Iraqi patients undescribed.

To address these known gaps, the present study characterized the sputum bacterial microbiome of Iraqi patients with bronchiectasis and healthy controls, using 16S rRNA gene (V3-V4) amplicon sequence (ASV)-based analysis. We characterized the taxonomic composition, evaluated alpha and beta diversity, and identified the differentially abundant bacterial genera between groups. In addition, we predicted the functional potential (KEGG pathways) for both communities using Tax4Fun2. Studying the taxonomic and predicted functional potential in a previously unstudied Iraqi cohort, this study provides the first characterization of the bronchiectasis sputum microbiome in Iraq and expands current understanding of the geographical variability of airway microbial communities in bronchiectasis.

## 2 Materials and methods

### 2.1 Subject recruitment

This cross-sectional study included adults (≥ 18 years) with a physician-confirmed diagnosis of non-cystic fibrosis bronchiectasis who attended AL-Yarmouk Teaching Hospital and the National Tuberculosis Institute between January 2023 and July 2024. The diagnosis of bronchiectasis had been established by a treating respiratory physician based on high-resolution computed tomography (HRCT) findings documented in the patient's medical records. Patients were eligible if they were able to provide a spontaneous sputum specimen for microbiological and microbiome analysis. Patients with other chronic respiratory diseases, severe immunosuppression, treatment with systemic glucocorticoids or immunosuppressive agents within the preceding 3 months, unavailable microbiological culture results, or inadequate sputum specimens were excluded from this study. Healthy controls were adults without a history of bronchiectasis or other chronic respiratory diseases. All controls were non-smokers and had no known chronic medical conditions. The study was approved by the Ministry of Higher Education and the Ministry of Health (Approval ID: 10609–28/2/2024), and written informed consent was obtained from all participants before enrollment.

### 2.2 Specimen collection and processing

Sputum samples were collected as spontaneous expectorations. Patients were instructed to cough deeply from the chest and expectorate sputum into a sterile universal container, while avoiding nasal secretions or saliva. Each sputum sample was stored at −80 °C until microbiota sequencing. Routine Ziehl-Neelsen staining was performed as part of the clinical microbiological assessment. No formal cytological quality assessment of sputum samples was performed.

### 2.3 Total bacterial DNA extraction

Total bacterial genomic DNA was extracted from sputum samples using the ReliaPrep™ Blood gDNA Miniprep System Kit (Promega USA), according to the manufacturer's instructions.

### 2.4 DNA quantification

DNA concentration was measured using a Quantus Fluorometer (Promega, USA). Briefly, 1 μl of genomic DNA was mixed with 199 μl of diluted Quantifluor Dye, and the mixture was incubated for 5 min at room temperature, and the fluorescence was measured according to the manufacturer's instructions.

### 2.5 Primer preparation

The lyophilized primers (Macrogen Inc., Seoul, South Korea) were dissolved in nuclease-free water to a final concentration of 100 pmol μl^−1^ as a stock solution according to the manufacturer's instructions.

### 2.6 16S rRNA gene (V3-V4) amplicon library preparation and sequencing

PCR amplification of the V3-V4 hypervariable regions of 16S rRNA was performed using the specific primer pair listed in Table 1^17^ and applied to Illumina sequencing-adapter overhang according to the Illumina16S Metagenomic Sequencing Library Preparation protocol. https://support.illumina.com/documents/documentation/chemistry_documentation/16s/16s-metagenomic-library-prep-guide-15044223-b.pdf. The PCR reaction was performed using 2× Go Taq Green Master mix with the following cycling conditions: 95 °C for 3 min; 25 cycles of 95 °C for 3 s, 55 °C for 30s, and 72 °C for 30s, and a final extension at 72 °C for 5 min. Indexed libraries were pooled and sequenced on an Illumina iSeq 100 platform, generating paired-end 150 bp reads (2 × 150 bp).

**Table 1:** Primers used in this study.

Primer	Nucleotide Sequence ^a^	Annealing Temp.(°C)	Product Size (bp)	Reference
16S amplicon PCR forward primer ^b^	5՛-TCGTCGGCAGCGTCAGATGTGTATAAGAGACAG-CCTACGGGNGGCWGCAG-՛3	55	550	17
16S amplicon PCR Reverse primer ^b^	5՛-GTCTCGTGGGCTCGGAGATGTGTATAAGAGACAG-GACTACHVGGGTATCTAATCC-՛3			

**a**. International Union of Pure and Applied Chemistry (IUPAC) nucleotide nomenclature: N = any base; W = A or T; H = A or C or T; V = A or C or G.

**b**. The primer sequence before the hyphen is the Illumina overhang adapter sequence. The primer sequence after the hyphen corresponds to the locus-specific sequence.

### 2.7 Sequencing processing and amplicon sequence variant (ASV) inference

Sequencing generated paired-end 150 bp reads (2 × 150 bp) using the Illumina iSeq 100 platform. The raw paired-end FASTQ files from all 38 sputum samples were processed in R (v4.6.1) using the DADA2 package (v1.40.0).^18^ Primer sequences were removed by trimming a fixed 17 bp (forward) and 21 bp (reverse)from the 5′ end for each read. Reads were quality-filtered; maxEE = 5, truncQ = 2, maxN = 0, and rm.phix = TRUE, retaining a mean of 97.4% of reads per sample. Amplicon sequencing variants were inferred from forward reads only because, after quality trimming, the forward and reverse reads showed only 10 bp of overlap across the ∼460 bp V3-V4 amplicon, which was insufficient for reliable read merging. Error-rate learning, sample inference, and ASV construction were performed using the standard DADA2 workflow. Chimeric sequences were removed, and the final ASV table was generated for 38 samples, providing 80 ASVs and 20,223 reads.

### 2.8 Taxonomic assignment

Taxonomy was assigned to each ASV using a naïve Bayesian classifier implemented in DADA2 against the SILVA reference database (v138.2). The species-level assignment was performed by exact sequence matching using the addSpecies () function in DADA2 17, 18]. The ASV count table was filtered to remove low-abundance ASVs and normalized using Total Sum Scaling (TSS) before downstream analysis. Of the 80 inferred ASVs, 76 (95%) were successfully classified to the genus level, representing 7 bacterial phyla and 24 unique genera, while 9 AVSs were assigned to the species level by exact sequence matching.

### 2.9 Construction of phylogenetic tree

The phylogenetic tree was constructed directly in R using the inferred ASV sequences. Multiple-sequence alignment was conducted using the DECIPHER package (v3.7.1).^19^ An initial neighbour-joining tree was generated and optimized using a maximum-likelihood framework using the phangorn package (v2.121) with the general time-reversible model incorporating invariant sites (GTR + I).^20^ The resulting tree was visualized as an annotated circular phylogram using ggtree (v4.2.0)^21^ and ggtreeExtra (v1.22.1).^22^ Concentric annotation rings were added to display phylum-level taxonomy, mean relative abundance, and genus-level ALDEx2 effect size, illustrating differences in the relative abundance of bacterial genera between bronchiectasis patients and healthy individuals.

### 2.10 Diversity analysis

Alpha diversity was assessed using observed ASVs (richness), Chao1 richness estimator, Shannon index, Simpson index, and Faith's phylogenetic diversity (PD). Diversity metrics were calculated using the vegan package (v2.7.5) and the picante package (v1.8.2). Beta diversity was assessed using Bray-Curtis dissimilarity, Jaccard distance, and weighted and unweighted UniFrac distance matrices was calculated with the GUniFrac package (v 1.9).^23^ Community dissimilarities were visualized by principal coordinates analysis (PCoA). Differences in alpha diversity between groups were evaluated using the Wilcoxon rank-sum test, whereas differences in beta diversity were assessed using permutational multivariate analysis of variance (PERMANOVA).

### 2.11 Differential abundance analysis

The ASV counts were assigned to the genus level before differential abundance was analysed. The differential abundance between patients and healthy groups was identified using ALDEx2 (v1.44.0).^24^ ALDEx2 was performed using 128 Monte Carlo instances to account for the compositional nature of microbiota data. Statistical significance was evaluated using Welch's *t*-test and the Wilcoxon rank-sum test with *P* values adjusted for multiple comparisons using the Benjamini-Hochberg (BH) false discovery rate (FDR) correction.

### 2.12 Functional prediction

The functional potential of the bacterial communities was predicted from 16S rRNA gene profiles using Tax4Fun2 (v1.1.5)^25^ with the Ref99 reference database. The representative ASV sequences were aligned to the reference database using local BLASTn (v2.16.0) with a minimum sequence similarity threshold of 97%.^26^ Tax4Fun2 was used to infer KEGG orthologs (KOs) and reconstruct predicted KEGG metabolic pathways. The predicted functional pathway abundances were compared between two groups using the Wilcoxon rank-sum test at *P* values adjusted for multiple comparisons using BH false discovery rate (FDR) correction. All statistical analyses were performed in R (v4.6.1).

## 3 Results

### 3.1 Study population

A total of 38 sputum samples were included in this study, comprising 28 samples from patients with bronchiectasis (73.6%) and 10 samples from healthy individuals (26.3%). The sex distribution differed between the two groups. Among patients with bronchiectasis, 17 (60.7%) were female, and 11(39.3%) were male, whereas among healthy controls, 4 (40.0%) were female, and 6 (60.0%) were male. The distribution of participants across the recorded age categories and other demographic characteristics is presented in (Table 2).

**Table 2:** General features of the groups included in this study.

Characteristics	Bronchiectasis (*n* = 28)	Health controls (*n* = 10)
Male n (%)	11 (39.3)	6 (60.0)
Female n (%)	17 (60.7)	4 (40.0)
Age group (Year)	21–41	41–61
Smoking status	Not systematically reported	Non-smoker
Chronic diseases	Not systematically reported	None reported

n = number of male and female patients.

### 3.2 Sequencing output and ASV inference

A total of 28,148 raw paired-end reads were obtained from 38 sputum samples, consisting of 28 patients with bronchiectasis and 10 healthy individuals, with a mean of 741 reads per sample (range 462–1005). A total of 27,394 reads (97.4%) were retained for downstream analysis after primer trimming and quality filtering. AVSs were inferred from forward reads, resulting in a final dataset of 80 ASVs represented by 20,223 high-quality reads following chimera removal. Taxonomic analysis yielded 7 bacterial phyla and 24 bacterial genera.

### 3.3 Taxonomic composition of the microbiota associated with bronchiectasis

**Fig. 1:**
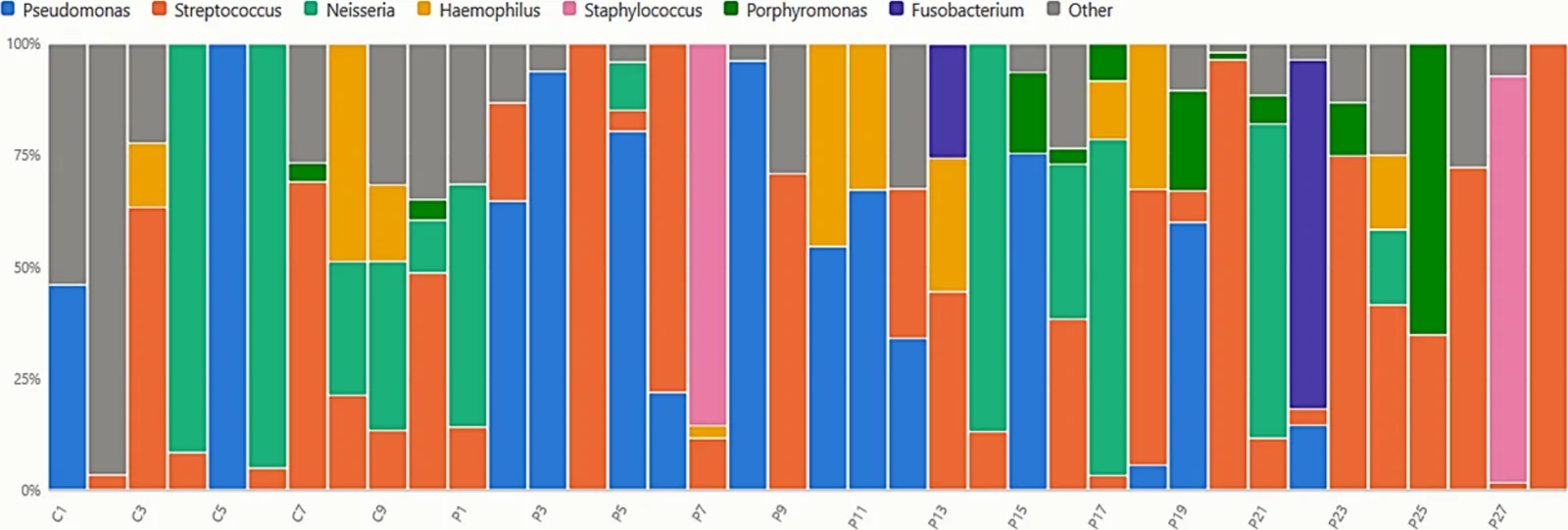
Genus-level taxonomic composition of sputum microbiota in bronchiectasis patients and healthy controls. Each bar represents one sample, and only the 7 most abundant genera are shown individually. The remaining genera are grouped as (Other). Relative abundances are based on ASV assignments generated by the DADA2 pipeline and taxonomically assigned against the SILVA v138.2 reference database.

Taxonomic profiling identified 24 bacterial genera belonging to 7 bacterial phyla across 38 sputum samples. Overall, the sputum microbiota was dominated by the genera *Pseudomonas* (mean relative abundance 25.4%), *Streptococcus* (23.9%), *Neisseria* (17.6%), *Haemophilus* (7.6%), and *Staphylococcus* (5.5%). At the genus level, numerical differences in mean relative abundance were observed between bronchiectasis and healthy groups. The patient group exhibited higher mean relative abundance of *Pseudomonas* (29.3%), *Staphylococcus* (7.7%), *Porphyromonas* (5.1%), and *Fusobacterium* (4.9%), whereas the healthy group showed higher relative abundance of *Neisseria* (28.0%), *Haemophilus* (8.6%), *Serratia* (8.2%), and *Enterobacter* (4.7%), and *Prevotella* (3.7%) (Fig. 1). These differences describe the relative abundance profiles; statistical evaluation of differential abundance is presented separately in section 3.7.

### 3.4 Phylogenetic relationships of the identified ASVs

**Fig. 2:**
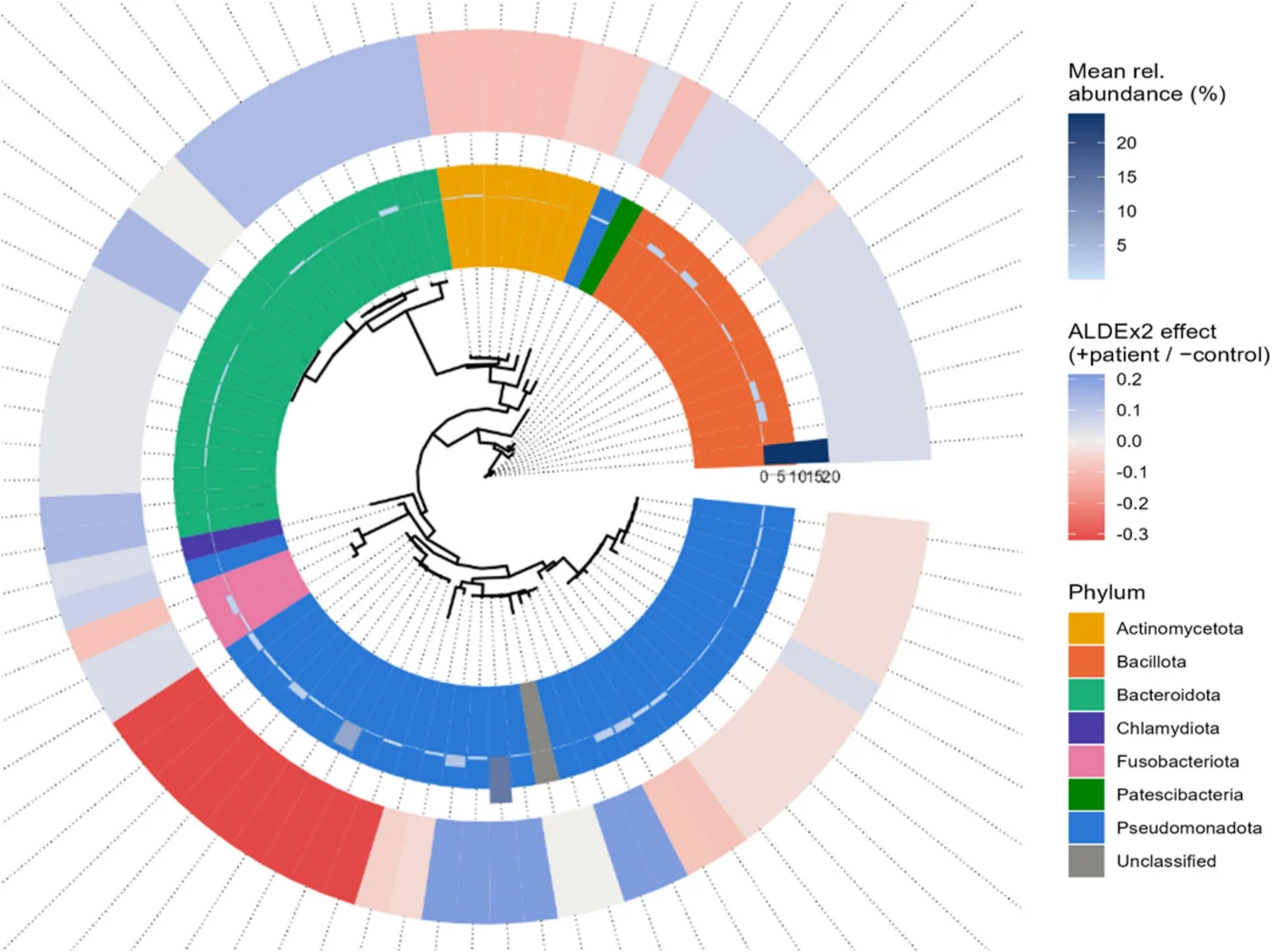
Maximum-likelihood phylogenetic tree of the 80 amplicon sequence variants (ASVs) identified from sputum samples. The circular phylogram integrates phylogenetic relationships with phylum-level classification, mean relative abundance (%), and genus-level ALDEx2 effect sizes comparing bronchiectasis patients and healthy controls. Together, these annotations provide a comprehensive overview of the taxonomic composition and phylogenetic structure of the sputum microbiota.

A maximum-likelihood phylogenetic tree was constructed from the 80 inferred ASVs to illustrate their evolutionary relationships and taxonomic distribution (Fig. 2). The identified ASVs were assigned to 7 bacterial phyla, with the majority belonging to Pseudomonadota, Bacillota, and Bacteroidetes. The circular phylogram integrates phylogenetic relationships with phylum-level classification, mean relative abundance, and genus-level ALDEx2 effect sizes, providing a comprehensive overview of the taxonomic composition and distribution of the sputum microbiota across the study cohort.

### 3.5 Alpha diversity analysis

**Fig. 3:**
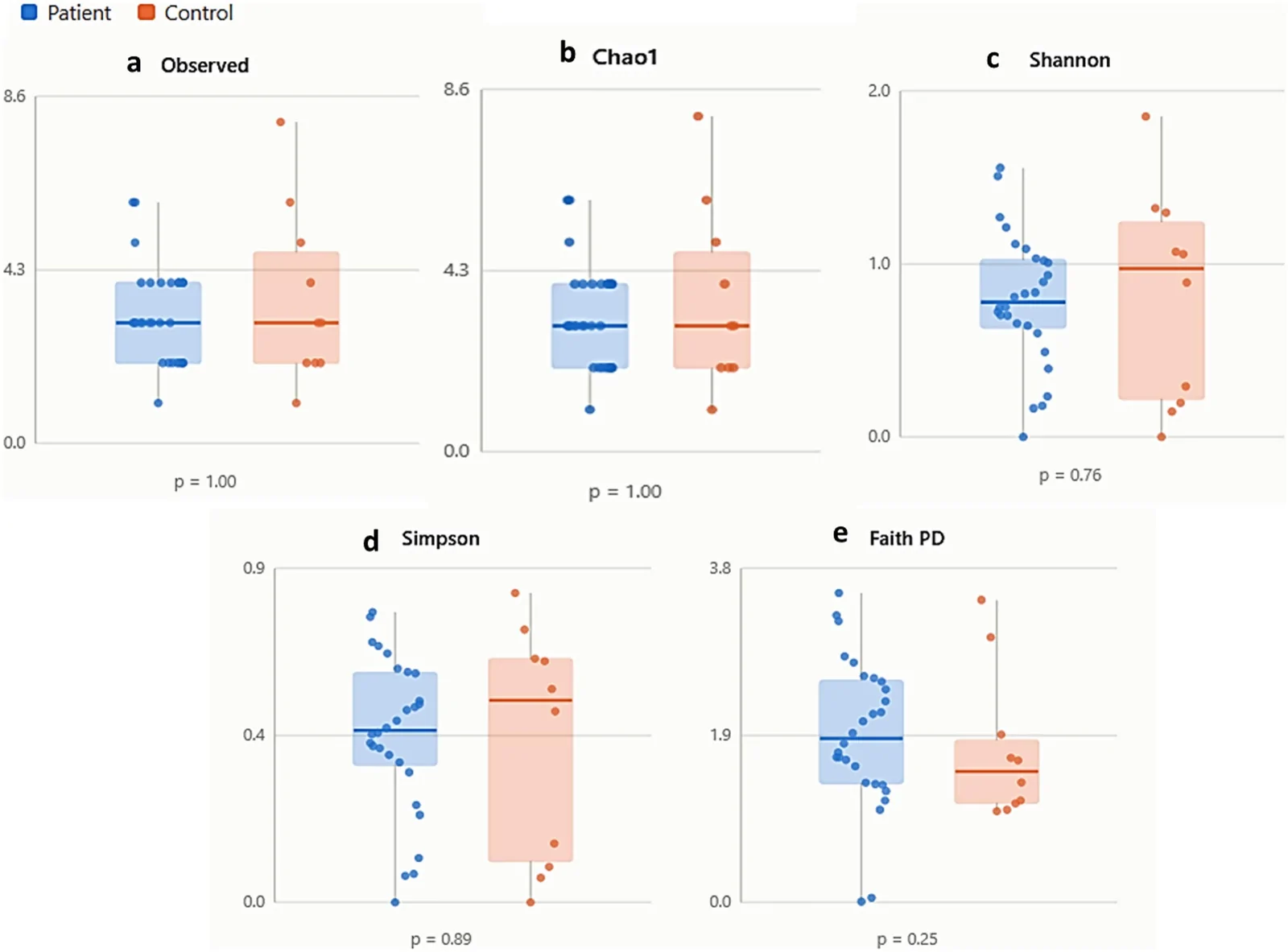
Alpha diversity of the sputum microbiota in patients with bronchiectasis and healthy controls. Alpha diversity was evaluated using: a: Observed ASV richness; b: Chao1; c: Shannon diversity index; d: Simpson index; e: Faith PD. Boxplots represent the median and interquartile range, with individual points corresponding to individual sputum samples. Differences between groups were assessed using the Wilcoxon rank-sum test. No statistically significant differences were observed for any diversity metrics.

The alpha diversity of the sputum microbiota was analysed by observed ASV richness, Chao1, Shannon diversity, Simpson diversity, and Faith's phylogenetic diversity (Fig. 3). The mean observed richness across all samples was 3.34 ASVs per sample (range of 1–8). All alpha diversity metrics showed no statistically significant differences between patient and healthy groups, including Observed (*p* = 1.00), Chao1 (*p* = 1.00), Shannon (*p* = 0.76), Simpson (*p* = 0.89), and Faith PD (*p* = 0.25) (Figure_3 a, b, c, d, e).

### 3.6 Beta diversity

**Fig. 4:**
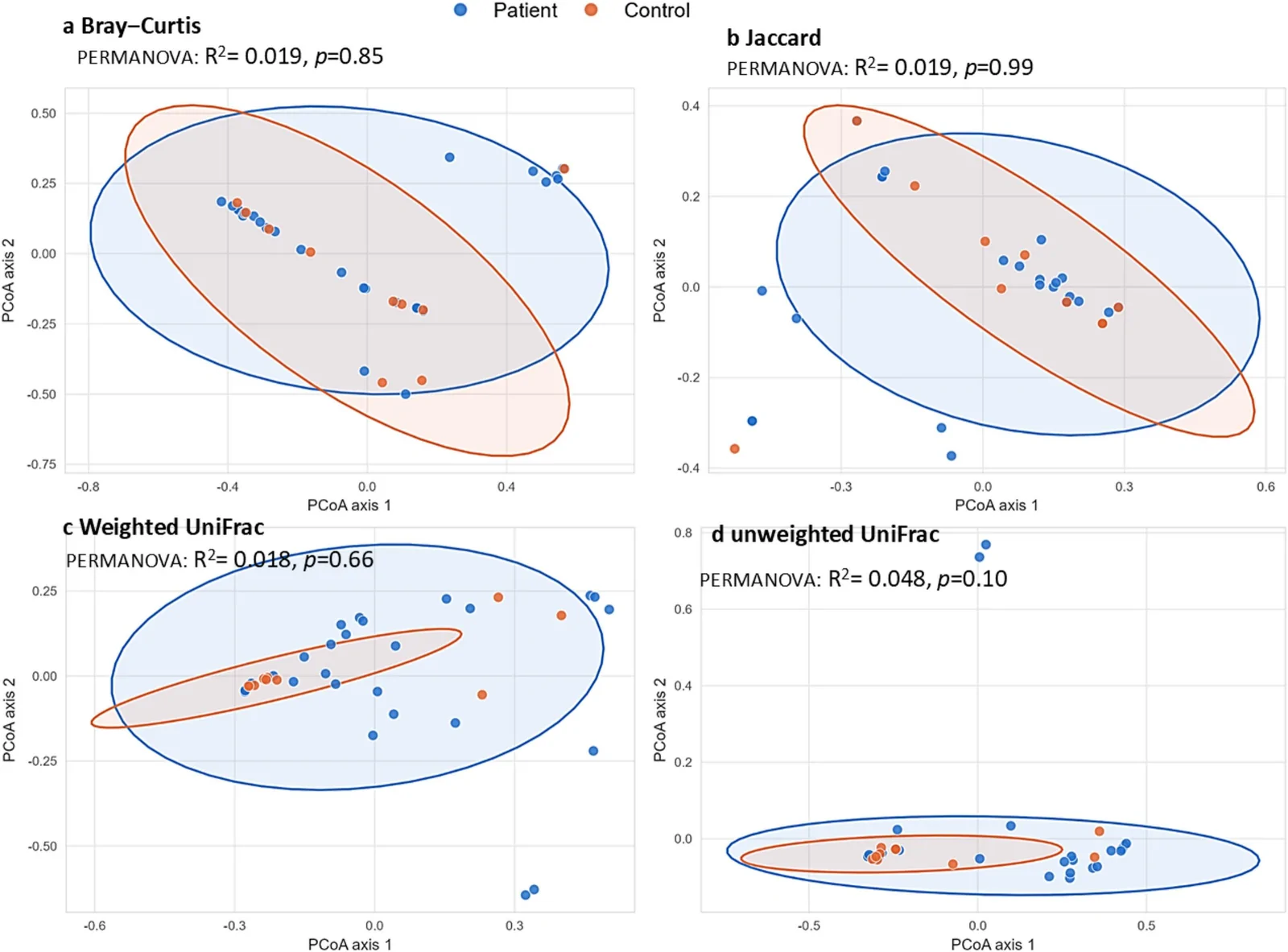
Beta diversity of sputum microbiota in patients with bronchiectasis and healthy controls. Principal coordinates analysis (PCoA) was performed using: a: Bray-Curtis dissimilarity, b: Jaccard distance, c: weighted UniFrac, and d: unweighted UniFrac distance metrics. Each point represents an individual sputum sample, and the shaded ellipses indicate the 95% confidence region for each study group. Group differences were evaluated using PERMANOVA. No significant differences in community composition were observed between the two groups across any of the four distances.

Beta diversity of the sputum microbiota was evaluated using Bray-Curtis dissimilarity, Jaccard distance, Weighted UniFrac, and Unweighted UniFrac metrics and visualized by principal coordinate analysis (PCoA) (Fig. 4). Across all distance metrics, the microbial communities of patients and controls showed substantial overlap, with no significant clustering according to the disease status. PERMANOVA analysis confirmed the absence of significant differences between groups for Bray-Curtis (*R*^*2*^ = 0.019, *p* = 0.85); Jaccard (*R*^*2*^ = 0.019, *p* = 0.99); weighted UniFrac (*R*^*2*^ = 0.018, *p* = 0.66), and unweighted UinFrac (*R*^*2*^ = 0.048, *p* = 0.100).

### 3.7 Differential abundance analysis

**Fig. 5:**
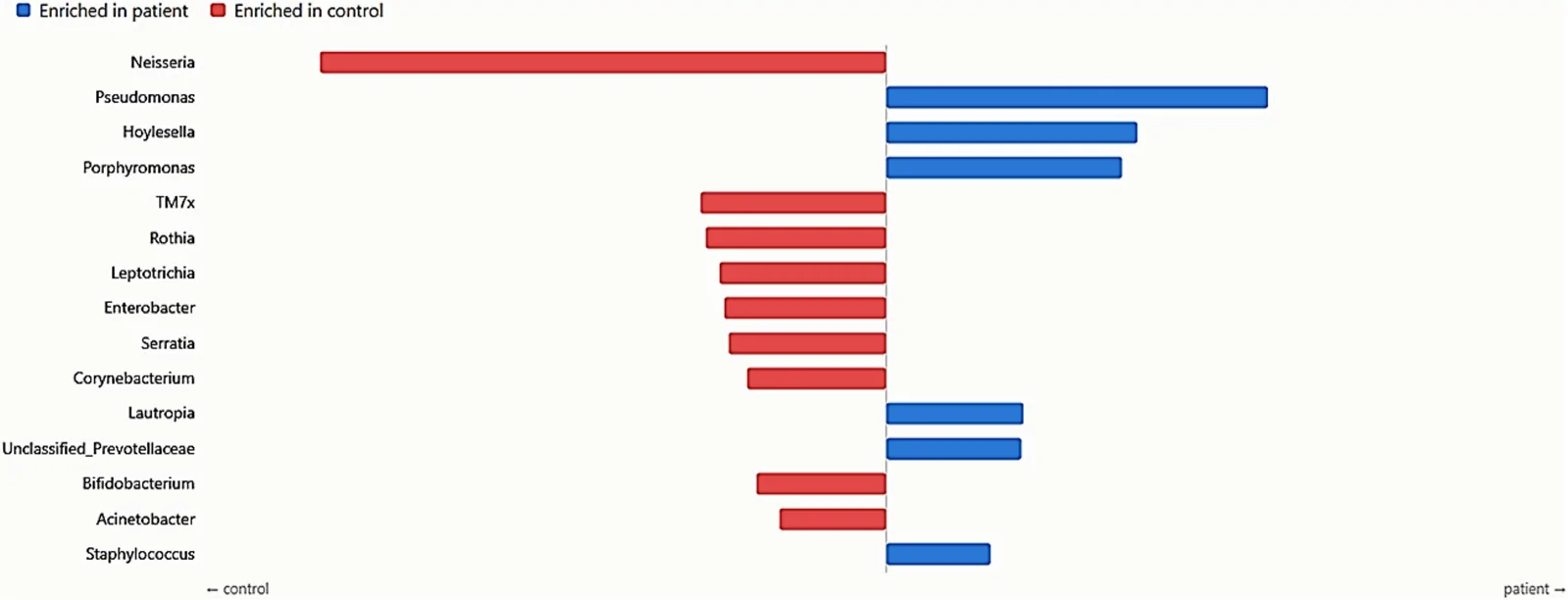
Differential abundance analysis of bacterial genera using ALDEx2. Genus-level effect sizes comparing bronchiectasis patients and healthy controls are shown for the 15 genera with the largest absolute effect sizes. Positive effect sizes indicate genera with high relative abundance in bronchiectasis, whereas negative effect sizes indicate genera with higher relative abundance in healthy controls. None of the analysed genera remained statistically significant after BH correction (*q* > 0.05).

Differential abundance analysis was performed on 27 bacterial genera using ALDEx2. None of the examined genera remained statistically significant after BH correction for multiple testing (all *q* *≥* 0.05), and none reached the pre-defined effect-size thresholds (|effect| ≥ 1) (Fig. 5). The largest positive effect sizes were observed for *Pseudomonas* (effect = 0.22), *Hoylesella* (effect = 0.15) and *Porphyromonas* (effect = 0.14), indicating higher relative representation in bronchiectasis patients. In contrast, *Neisseria* showed the largest negative effect size (effect = −0.32), indicating a higher relative abundance in the control group.

### 3.8 Predicted functional profile

**Fig. 6:**
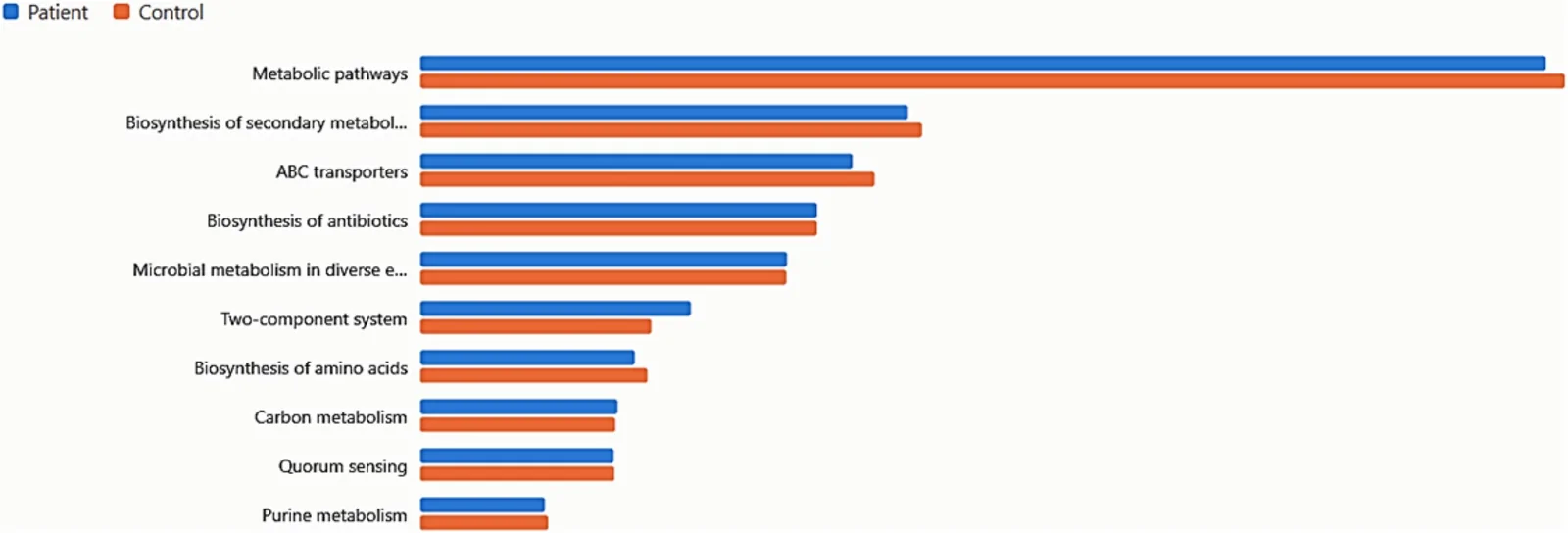
Predicted functional profile of the sputum microbiota based on KEGG pathway analysis using Tax4Fun2. Bars represent the mean relative abundance of the ten most abundant KEGG pathways in patients with bronchiectasis and healthy controls. Four pathways showed nominal differences between groups (*p* < 0.05), but none remained significant after BH correction (all *q* = 1.0).

The functional potential of the sputum microbiota was predicted using Tax4Fun2 based on KEGG pathways. The predicted functional profile was dominated by broad bacterial metabolic functions, including metabolic pathways (14.4% mean relative abundance), biosynthesis of secondary metabolites (6.2%), ABC transporters (5.6%), Biosynthesis of antibiotics (5.0%), and two-component systems (3.3%) (Fig. 6).

Among 305 predicted KEGG pathways, 4 pathways showed nominal differences between patients with bronchiectasis and healthy controls (Wilcoxon *p* < 0.05): linoleic acid metabolism, monobactam biosynthesis, nicotinate and nicotinamide metabolism, and penicillin/cephalosporin biosynthesis. However, none remained statistically significant after BH false discovery rate (BH *q* = 1.0 for all).

## 4 Discussion

To our knowledge, this is the first study to characterize bacterial microbiome in sputum of Iraqi patients with bronchiectasis using 16S rRNA gene (V3-V4) amplicon sequencing. Taxonomic profiling revealed that the sputum microbiome was dominated by *Pseudomonas*, *Streptococcus, Neisseria* and *Haemophilus.* Although differences in the relative abundance of several genera were observed between bronchiectasis patients and healthy controls, alpha diversity, beta diversity, differential abundance after multiple-testing correction, and predicted functional pathways did not differ significantly between groups. These findings suggest that the overall bacterial community structure was largely similar between the study groups, while certain taxa showed trends that warrant further investigation in larger cohorts.

The core airway microbiota includes bacterial genera such as *Veillonella*, *Haemophilus*, *Pseudomonas*, and *Streptococcus*, whose relative abundances vary among individuals and clinical conditions.^27^ In the present study, *Pseudomonas* showed a higher mean relative abundance in patients with bronchiectasis (29.3%), whereas *Neisseria* showed a higher mean relative abundance in healthy controls (28.0%). These differences were descriptive and did not remain statistically significant after correction of multiple comparisons. Previous studies have reported differences in the composition and diversity of sputum microbiota between bronchiectasis patients and healthy individuals in Asia and Uganda.^28^, ^29^ In contrast, some studies of respiratory disease microbiota communities have identified relatively limited microbial community profiles.^30^, ^31^ Differences among studies may reflect variation in study populations, clinical characteristics, disease severity, geographical setting, sample sizes, and methodological approach. In addition, most commensal bacteria dominate in patients and the healthy population, which may suggest potential resilience or resistance of the commensal microbiota toward acute infection.^32^ In our study, the high relative abundance of *Pseudomonas* in patients reflects the abnormal growth of this pathogen,^33^, ^34^ and may be a key microbial determinant of gut–lung interaction in bronchiectasis patients.^35^

The prevalence of many *Pseudomonas* species in the lower respiratory tract may be due to a shift in the lower airways and lungs, which is suitable for this pathogen.^36^ Moreover, *Streptococcus*, *Prevotella* and *Veillonella* are the most abundant genera in the healthy population in China and Uganda.^37^ Nevertheless, a study reported that *Streptococcus*, *Neisseria*, and *Veillonella* are the most prevalent genera in Indian patients.^29^ Although several dominant genera identified in the present study have also been reported in previous bronchiectasis microbiome studies, differences in their relative abundance and community structure are expected. These differences may be due to geographical location variations, environmental exposure, dietary habits, disease aetiology, severity of disease, antibiotic exposure, sputum collection and processing strategy, sequencing platform, bioinformatic analysis, and reference databases used for taxonomic assignment.^3^, ^6^ Several genera, such as *Bacillus, Anoxybacillus, Granulicatella, Stenotrophomonas, Sphingomonas, Fervidicoccus, and Lactobacillus*, have been reported as constituents of the sputum microbiota in pulmonary-related diseases in India and China^28^, ^29^; however, these genera were not detected in our study. These differences may be due to inherent environmental characteristics in different geographical regions.^37^ Moreover, differences in sample size and patient selection criteria among studies may contribute to the observed variability in microbial composition. In general, these factors emphasize the heterogeneous nature of the bronchiectasis airway microbiome and highlight the importance of regional studies to better understand population-specific microbial profiles.^5^

Alpha diversity metrics (observed ASV richness, Chao1, Shannon diversity, Simpson diversity, and Faith's phylogenetic diversity) revealed no significant differences in microbiome composition between the two groups.^38^ Our results are supported by those of previous studies.^39^ In contrast, few studies have reported an increase in the alpha diversity of the lung microbiome.^40^, ^41^ Beta diversity analysis (Bray-Curtis dissimilarity, Jaccard distance, Weighted UniFrac, and Unweighted UniFrac metrics) showed no statistically significant differences (Bray-Curtis (*R*^*2*^ = 0.019, *p* = 0.85); Jaccard (*R*^*2*^ = 0.019, *p* = 0.99); weighted UniFrac (*R*^*2*^ = 0.018, *p* = 0.66), and unweighted UinFrac (*R*^*2*^ = 0.048, *p* = 0.100). An overall reduction in the diversity of the sputum microbiota associated with bronchiectasis and overgrowth of certain pathogenic taxa was previously reported.^42^

The differential abundance revealed that none of the examined genera remained statistically significant. The largest positive effect sizes were observed for *Pseudomonas* (effect = 0.22) in bronchiectasis patients, indicating a potential contribution of these genera to respiratory disease exacerbation.^43^

The predicted functional profile was dominated by broad bacterial metabolic functions, including metabolic pathways (14.4% mean relative abundance), biosynthesis of secondary metabolites (6.2%), ABC transporters (5.6%), Biosynthesis of antibiotics (5.0%), and two-component systems (3.3%). Among 305 predicted KEGG pathways, 4 pathways showed nominal differences between patients with bronchiectasis and healthy controls (Wilcoxon *p* < 0.05): linoleic acid metabolism, monobactam biosynthesis, nicotinate and nicotinamide metabolism, and penicillin/cephalosporin biosynthesis. However, none remained statistically significant after BH false discovery rate (BH *q* = 1.0 for all).

## 5 Conclusion

The study provides the first characterization of the sputum bacterial microbiome in Iraqi patients with bronchiectasis using 16S rRNA gene (V3-V4) amplicon sequencing. The sputum microbiota of both bronchiectasis and healthy controls was dominated by *Pseudomonas*, *Neisseria*, *Streptococci*, and *Haemophilus*, with descriptive differences in the relative abundance of several bacterial genera between groups. However, no statistically significant differences were observed in alpha diversity, beta diversity, differential genus abundance after multiple-testing correction, or predicted KEGG functional pathways. These findings suggest that although compositional trends were present, larger and well-balanced cohorts are required to determine whether these observations represent a reproducible microbiome signature associated with bronchiectasis.

This study has several limitations. Firstly, the relatively small and unequal group sizes may reduce the statistical power to detect subtle microbiome differences. In addition, several clinical variables may affect the airway microbiome, such as smoking history in patients, recent antibiotic exposure, disease stability at sampling, findings of sputum culture, and other systemic factors that may not be recorded. Moreover, functional profiles were predicted from 16S rRNA gene data rather than measured directly. Future longitudinal studies incorporating larger cohorts and complementary metagenomic and functional approaches are warranted to validate these findings and further investigate the airway microbiome in bronchiectasis.

## CRediT authorship contribution statement

**Maryam Hadi:** Writing – original draft, Resources, Methodology, Investigation, Funding acquisition, Formal analysis. **H. Halah Al-Haideri:** Writing – review & editing, Validation, Supervision, Software, Project administration, Data curation, Conceptualization.

## Funding

The authors declare that no funds, grants, or other support was received during the preparation of this manuscript.

## Declaration of competing interest

The authors declare the following financial interests/personal relationships which may be considered as potential competing interests: Halah Al-Haideri reports was provided by University of Baghdad. If there are other authors, they declare that they have no known competing financial interests or personal relationships that could have appeared to influence the work reported in this paper.

## Data Availability

The datasets generated and analysed during the current study are available upon request.
